# Diffusion-weighted imaging in identifying breast cancer pathological response to neoadjuvant chemotherapy: A meta-analysis

**DOI:** 10.18632/oncotarget.23195

**Published:** 2017-12-11

**Authors:** Wei Chu, Weiwei Jin, Daihong Liu, Jian Wang, Chengjun Geng, Lihua Chen, Xuequan Huang

**Affiliations:** ^1^ Department of Radiology, Wuxi Huishan District People's Hospital, Jiangsu Province, 214187, China; ^2^ Department of Radiology, Wuxi Second Traditional Chinese Medicine Hospital, Jiangsu Province, 214121, China; ^3^ Department of Radiology, Southwest Hospital, Third Military Medical University, Chongqing, 400038, China; ^4^ Department of Radiology, PLA No.101 Hospital, Wuxi, Jiangsu Province, 214044, China

**Keywords:** magnetic resonance imaging, diffusion-weighted imaging, neoadjuvant chemotherapy, breast cancer, meta-analysis

## Abstract

**Background:**

Diffusion-weighted imaging (DWI) is increasingly used to identify pathological complete responses (pCRs) to neoadjuvant chemotherapy (NAC) in breast cancer. The aim of the present study was to assess the utility of DWI using a pooled analysis.

**Materials and Methods:**

Literature databases were searched prior to July 2017. Fifteen studies with a total of 1181 patients were included. The data were extracted to perform pooled analysis, heterogeneity testing, threshold effect testing, sensitivity analysis, publication bias analysis and subgroup analyses.

**Result:**

The methodological quality was moderate. Remarkable heterogeneity was detected, primarily due to a threshold effect. The pooled weighted values were a sensitivity of 0.88 (95% confidence interval (CI): 0.81, 0.92), a specificity of 0.79 (95% CI: 0.70, 0.86), a positive likelihood ratio of 4.1 (95% CI: 2.9, 5.9), a negative likelihood ratio of 0.16 (95% CI: 0.10, 0.24), and a diagnostic odds ratio of 26 (95% CI: 15, 46). The area under the receiver operator characteristic curve was 0.91 (95% CI: 0.88, 0.93). In the subgroup analysis, the pooled specificity of change in the apparent diffusion coefficient (ADC) subgroup was higher than that in the pre-treatment ADC subgroup (0.80 [95% CI: 0.71, 087] vs. 0.63 [95% CI: 0.52, 0.73], *P* = 0.027).

**Conclusions:**

DWI may be an accurate and nonradioactive imaging technique for identifying pCRs to NAC in breast cancer. Nonetheless, there are a variety of issues when assessing DWI techniques for estimating breast cancer responses to NAC, and large scale and well-designed clinical trials are needed to assess the technique's diagnostic value.

## INTRODUCTION

Neoadjuvant chemotherapy (NAC) has been used as a standard treatment for both initially operable and initially inoperable locally advanced breast cancer [1]. Patients who achieve a pathologic complete response (pCR; defined as no residual tumour or a minimal residual tumour on histologic analysis) demonstrate significantly longer disease-free and overall survival rates [2]. The early prediction of outcome and identification of the pCR to NAC are important for individualised therapies and avoiding the use of additional toxic therapies and provide a greater chance of achieving a pCR [3, 4].

Determining how to predict the pCR to NAC accurately remains a challenging clinical problem with no consensus approach. Based on their quantitative and noninvasive characteristics, several imaging tools, such as magnetic resonance imaging (MRI), mammography, and ultrasound, are used to monitor tumour size change after NAC [5]. However, these imaging techniques, which focus on monitoring changes in morphological features, are unable to distinguish potential residual cancer from fibrotic scar tissue in a stable tumour [6]. These limitations have led many researchers to explore other functional techniques, such as positron emission tomography, quantitative perfusion MRI, magnetic resonance spectroscopy and diffusion-weighted imaging (DWI). Based on the diffusion of water molecules through tumour tissue, DWI is a new means of predicting tumour responses to treatment [7]. The Brownian motion of water molecules in cancer is restricted, which results in a decreased apparent diffusion coefficient (ADC) value. Previous studies [8–10] have shown that the ADC is highly negatively correlated with tumour cellularity and could be used to estimate the tumour pathological response to therapy.

Many studies [11–25] have reported the accuracy of DWI in predicting pathological responses to NAC in breast cancer against a histopathologic reference standard. However, the findings of these studies have been incongruent, as different DWI techniques have been used, and most of the sample sizes have been small. Therefore, we conducted a meta-analysis to assess the diagnostic performance of DWI for monitoring pathological responses to NAC in breast cancer.

## RESULTS

**Figure 1:**
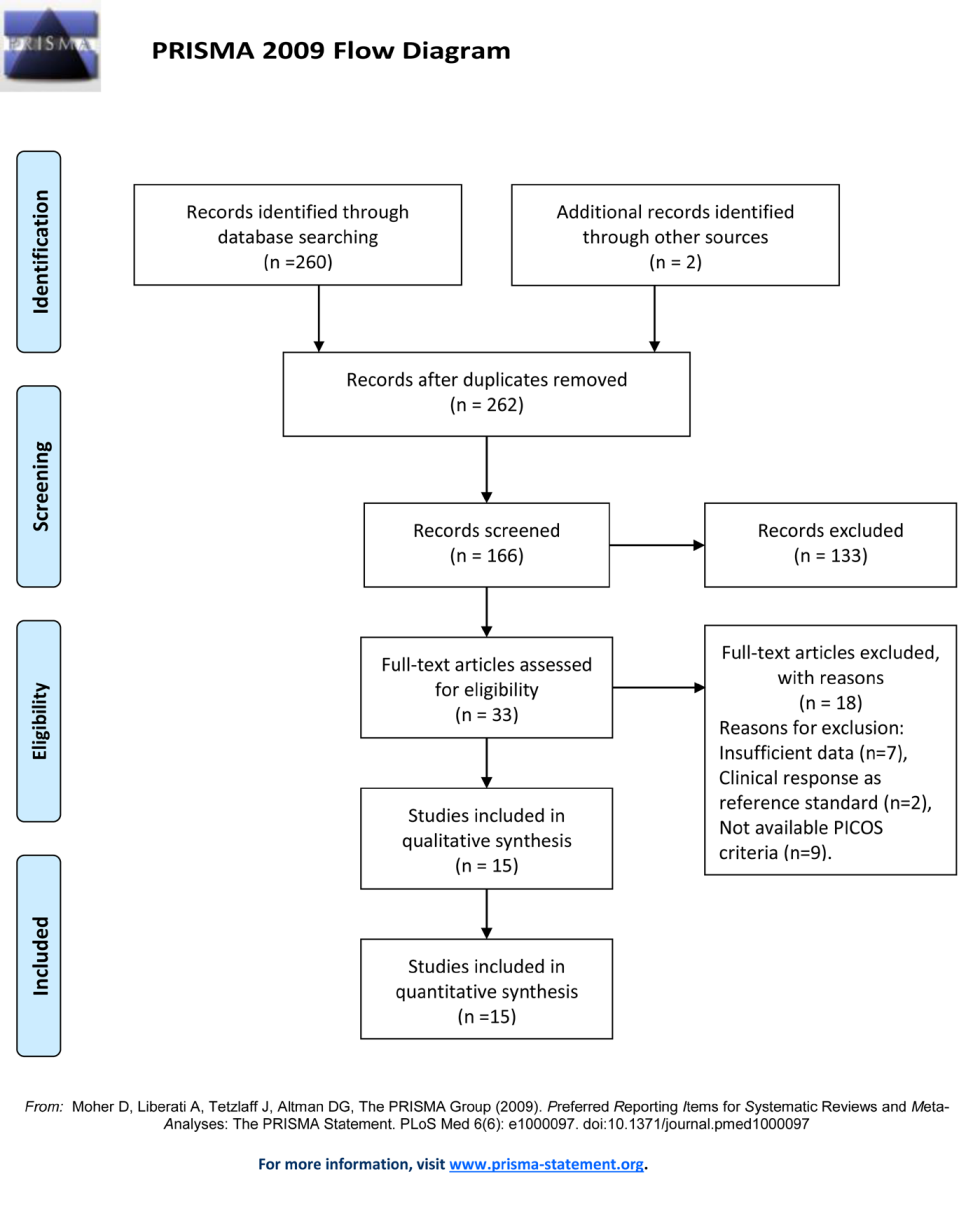
Flowchart illustrating the selection of the studies

Medical and scientific literature databases were searched and reference lists were cross-checked for original articles published prior to July 2017 (Figure 1), 166 articles were found in the primary result. There were 15 studies [11–25] with a total of 1181 breast cancer patients that met the inclusion criteria for quantitative synthesis.

The data extracted from these included studies are summarised in Tables 1, 2 and Supplementary Table 1. The mean number of patients per study was 74.6 (range 20–195), and the mean age of the patients in each study was 48.7 years (range 23–83). A variety of NAC regimens, definitions of pCR, MRI devices, DWI methods of measurement, analyses, and cutoff values were observed. Of the 15 studies, 9 studies [11–16, 22–24] evaluated both DWI and conventional contrast-enhanced MRI (CE-MRI) techniques in the same cohort, 6 studies [14, 16–18, 23, 25] were performed using 3T scanners, 5 studies [12–14, 22, 24] were performed with a b value ≥1000 s/m^2^, and 2 studies [14, 25] were performed using intravoxel incoherent motion (IVIM) models. There were 4 studies [12, 15, 16, 25] that enrolled patients prospectively, and 8 studies [11, 13, 17, 19–23] that enrolled patients retrospectively, while the remaining studies did not report this parameter. A total of 7 studies [11–13, 18, 19, 23, 24] used the change in ADC as a biomarker, while 3 studies [15, 21, 25] used the pre-treatment ADC, 2 studies [17, 20] used the post-treatment ADC, and 3 studies [14, 16, 22] used all three conditions.

**Table 1:** Cohort and tumour characteristics of the included studies

Variables		Studies No.	Patients No.	Mean	Range
**Cohort characteristics**					
**No., all tests**		15	1081	74.6	20–225
**pCR**		15	297	27.1%	12.9%-85.0%
**non-pCR**		15	783	72.9%	15.0%-87.1%
**Age (years)**		14	1011	48.7	23–83
**Tumour characteristics**					
**Stage**	II	7	173	31.5%	10.0%-63.3%
	III	8	355	64.5%	36.7%-100%
	IV	1	22	18.4%	-
**Histology**	IDC	12	799	88.0%	74.5%-97.2%
	ILC	10	85	9.8%	3.3%-22.6%
	MC	3	6	3.1%	2.9%-3.3%
	Other	8	33	4.2%	1.1%-11.7%
**Receptor**	ER (+)	6	133	32.7%	24.3%-46.9%
	PR (+)	5	134	32.8%	25.0%-39.1%
	HER-2 (+)	11	185	21.0%	6.3%-37.9%
	LA	4	109	26.5%	12.5%-38.1%
	LB	4	106	41.9%	25.6%-81.3%
	TN	7	138	19.5%	7.4%-33.9%

Note: ER = oestrogen receptor; PR = progesterone receptor; HER-2 = human epidermal growth factor receptor 2; IDC = invasive ductal carcinoma; ILC = invasive lobular carcinoma; LA = luminal A; LB = luminal B; MC = mucinous carcinoma; NAC = neoadjuvant chemotherapy; pCR = pathologic complete response; TN, triple negative.

**Table 2:** Principal characteristics of the included studies

Study	Year	Design	Time of scan	Field	Type	b value	Evaluate index	Cut-off	Sen	Spe
An, Y	2015	Retro	B/A(post NAC)	1.5T	DWI	0, 750	ΔADC	15.2%	0.67	0.71
Belli	2011	Pro	B/A(NR)	1.5T	DWI	0, 1000	ΔADC	68.0%	0.80	0.85
Bufi	2014	Retro	B/A(4–6 cycles)	1.5T	DWI	0, 1000	ΔADC	NR	0.87	0.59
Che	2016	NR	B/A(2–3 cycles)	3.0T	IVIM	0, 800	ΔD	Δ0.163^a^	1.00	0.79
							pre-D	0.874^a^	0.69	0.65
							post-D	0.971^a^	1.00	0.63
Fangberget	2011	Pro	B/A(4 cycles)	3.0T	DWI	100, 250, 800	pre-ADC	1.420^a^	0.91	0.81
Li	2015	Pro	B/A(1 cycles)	3.0T	DWI	0, 600	ΔADC	5.5%	0.50	0.76
							pre-ADC	1.2^a^	1.00	0.54
							post-ADC	1.4^a^	0.83	0.67
Liu	2015	Retro	B/A(post NAC)	3.0T	DWI	0, 800	post-ADC	NR	0.69	0.94
Luo	2014	NR	B/A(post NAC)	3.0T	DWI	0, 800	ΔADC	42.5%	0.89	0.74
Park	2010	Retro	B/A(post NAC)	1.5T	DWI	0, 750	post-ADC	1.17^a^	0.94	0.71
Park S	2012	Retro	B/A(3–6 cycles)	1.5T	DWI	0, 750	ΔADC	54.9%	1.00	0.70
Richard†	2013	Retro	B/A(post NAC)	1.5T	DWI	50, 700	pre-ADC	1.29^a^	1.00	0.38
Shin	2012	Retro	B/A(post NAC)	1.5T	DWI	100,500, 800,1000	ΔADC	40.7%	1.00	0.91
							pre-ADC	0.92^a^	0.80	0.65
							post-ADC	1.19^a^	1.00	0.70
Weis	2015	Retro	B/A(1 cycles)	3.0T	DWI	0, 500/600	ΔADC	NR	0.92	0.84
Woodhams	2010	NR	B/A(4 cycles)	1.5T	DWI	0, 1500	ΔADC	Category 1^b^	0.97	0.89
Bedair	2017	Pro	B/A(3 cycles)	3.0T	IVIM	0, 60, 120, 300, 600, 900	DDC	1.14^a^	0.79	0.73
							ADC	1.01^a^	0.79	0.67

Note: ^a^, ADC or D or DDC (×10^−3^ mm^2^/s); ^b^, DWI was classified into 4 categories: category 1 indicated no residual disease. †, the analysis was available in the triple-negative subtype.

Abbreviations: ADC, apparent diffusion coefficient; ΔADC, change in apparent diffusion coefficient; B/A , before NAC and after NAC; D, true molecular diffusion coefficient; DDC, distributed diffusion coefficient; ΔD, change in true molecular diffusion coefficient; IVIM, intravoxel incoherent motion; Pro, prospective; Retro, retrospective; NR, not reported; Sen, sensitivity; Spe, specificity.

**Figure 2:**
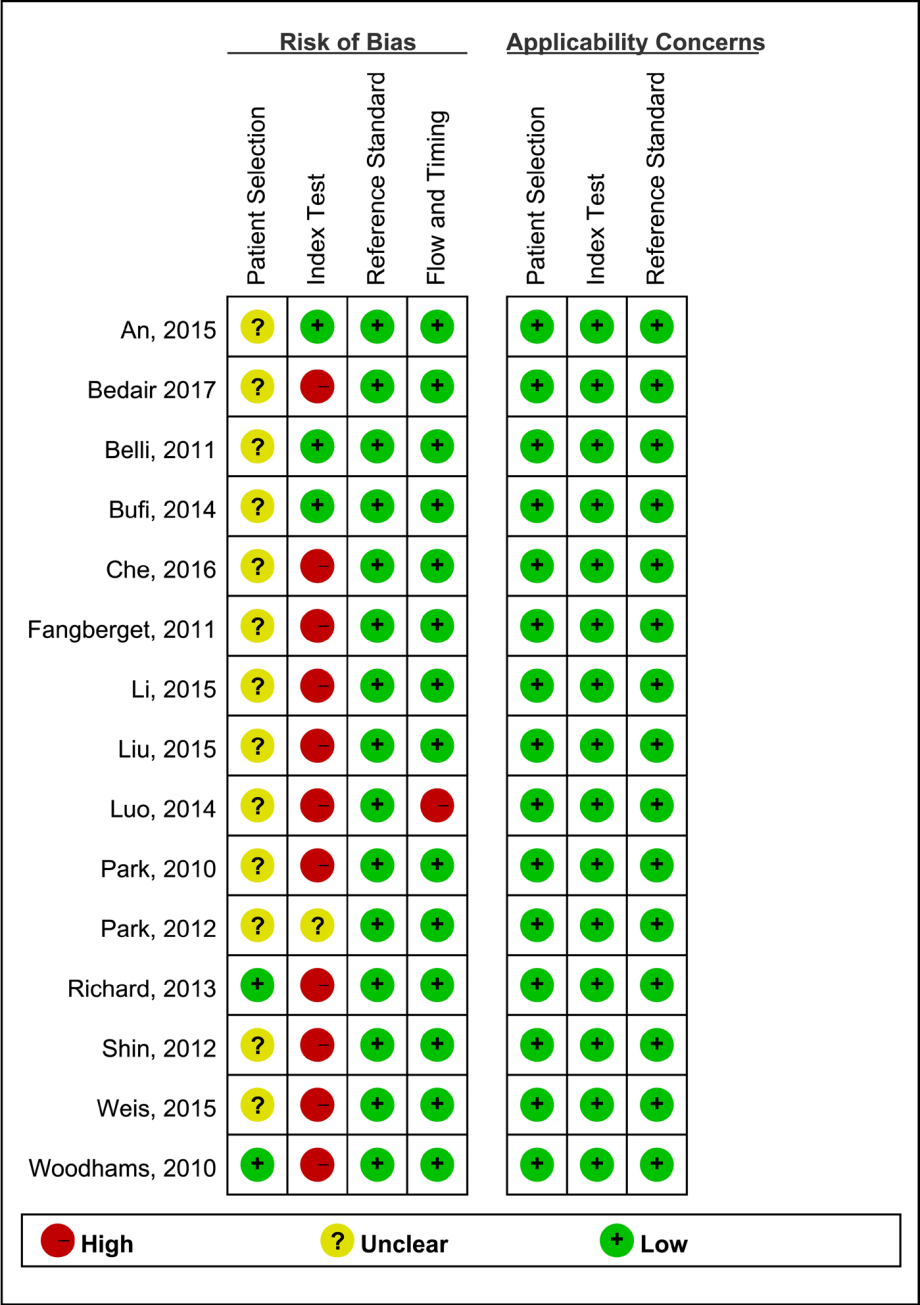
Risk of bias and applicability concerns summary of the 15 included studies

According to QUADAS-2 items, the quality assessment of 15 studies was moderate. Only 3 studies [11–13] set a pre-specified threshold. The results of the distribution are shown in Figure 2.

**Figure 3:**
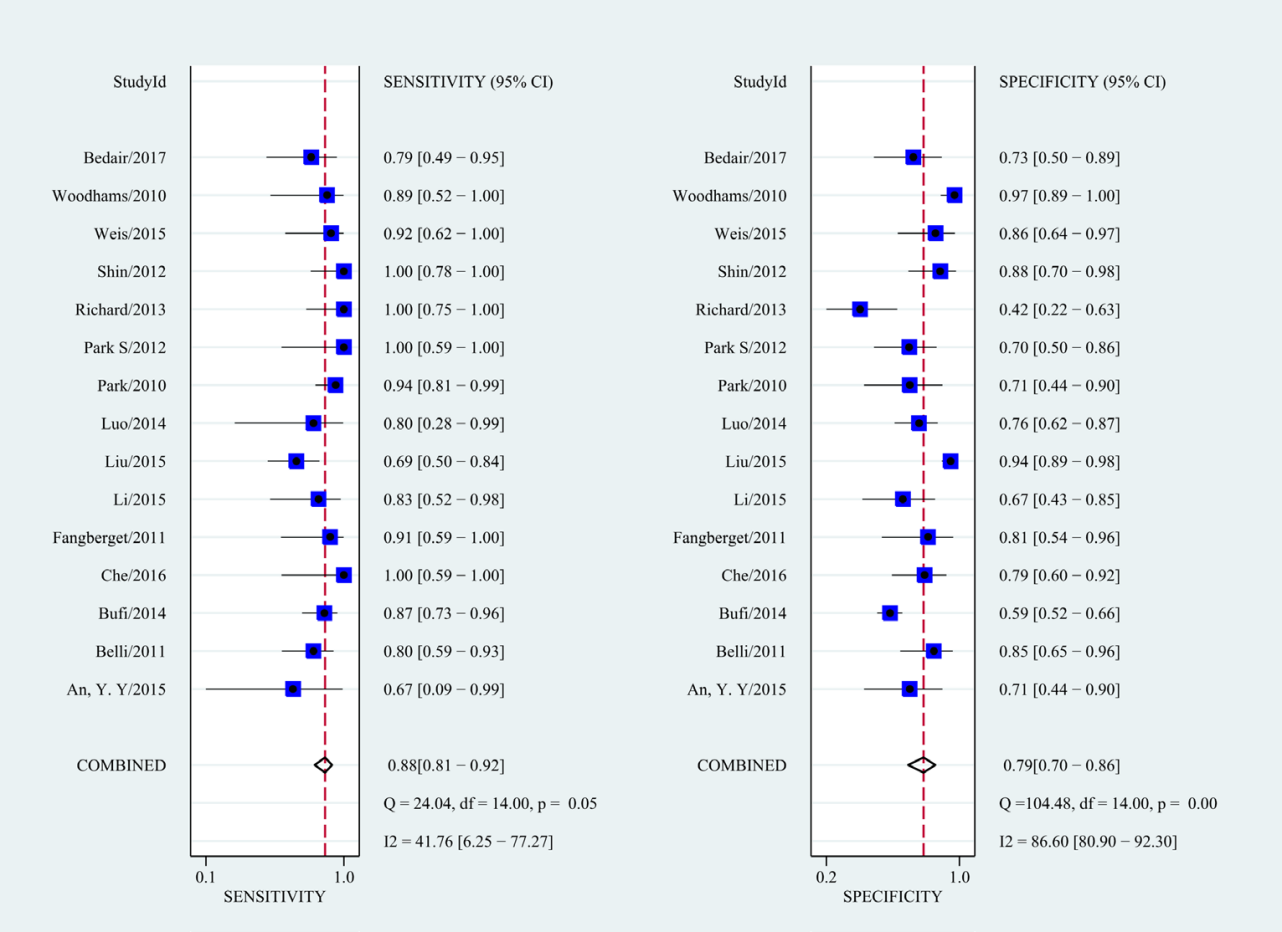
Forest plots of the SEN and SPE and corresponding 95% CIs for DWI as an assessor of the pathologic response to NAC

**Figure 4:**
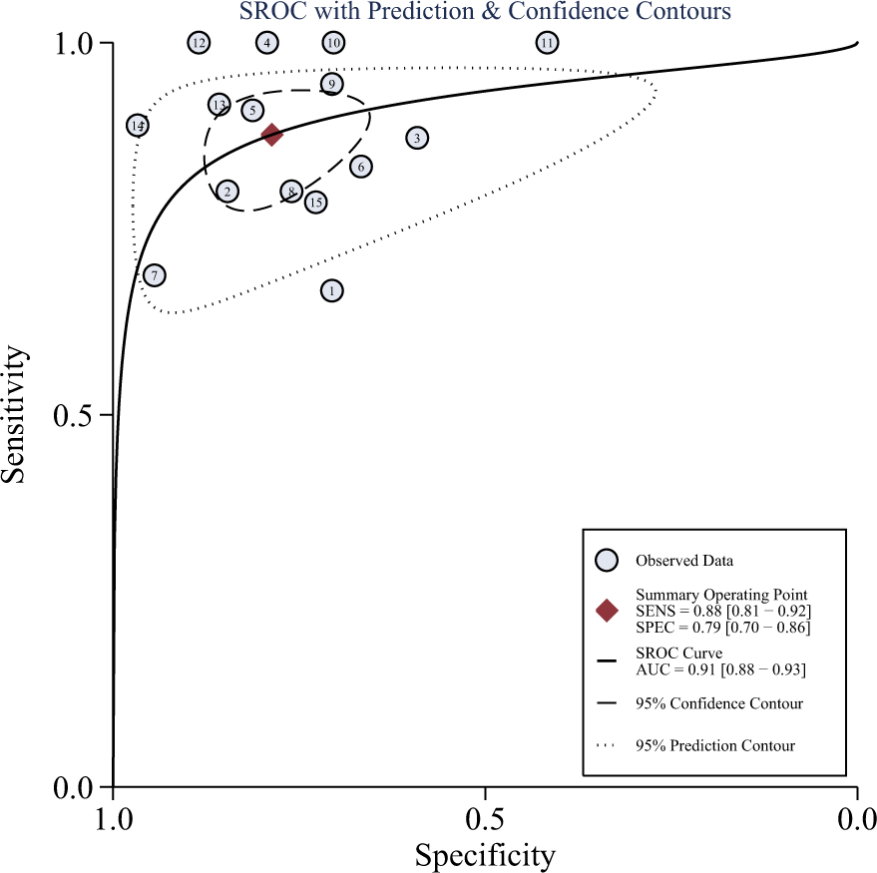
Hierarchical summary receiver operating characteristic (HSROC) curves from the bivariate model of DWI

As there was notable heterogeneity in the present meta-analysis (I^2^ = 93%, *P* < 0.001), we used a random-effects coefficient binary regression model. The pooled weighted values for DWI were a SEN of 0.88 (95% confidence interval (CI): 0.81, 0. 92), a SPE of 0.79 (95% CI: 0.70, 0.86), a positive likelihood ratio (PLR) of 4.1 (95% CI: 2.9, 5.9), an negative likelihood ratio (NLR) of 0.16 (95% CI: 0.10, 0.24), a diagnostic odds ratio (DOR) of 26 (95% CI: 15, 46), and an area under the ROC curve (AUC) of 0.91 (95% CI: 0.88, 0.93). Forest plots and HSROC curves of the 15 studies are shown in Figures 3, 4, Supplementary Figures 1, and 2. After the sensitivity analysis, 3 studies [17, 22, 24] were detected (Supplementary Figure 3). However, there was no effect on the results of the pooled weighted values when these studies were excluded. The proportion of heterogeneity likely due to a threshold effect was 95% in the accuracy estimates among individual studies. The results of meta-regression also indicated that b values, study design, MRI field strength, and DWI model were not strongly associated with accuracy.

The results of the subgroup analysis are presented in Table 3. In the subgroup analysis of b value, MRI field, study design and DWI model, no notable differences were observed. In the subgroup analysis of different biomarkers of DWI, the performance of the ΔADC subgroup was equivalent to that of the post-NAC ADC subgroup in assessing the pCR to NAC with comparable pooled sensitivity (0.88 [95% CI: 0.75, 0.94] vs. 0.91 [95% CI: 0.78, 0.96], P = 0.527) and pooled specificity (0.80 [95% CI: 0.71, 087] vs. 0.78 [95% CI: 0.58, 0.90], P = 0.398). However, the pooled specificity of theΔADC subgroup was higher than that of the pre-NAC ADC subgroup (0.80 [95% CI: 0.71, 087] vs. 0.63 [95% CI: 0.52, 0.73], *P* = 0.027).

**Table 3:** Sensitivity and specificity estimates for each subgroup

Subgroup	No. of studies	Mean SEN (%)	Mean SPE (%)	DOR	AUC (%)
b value (s/m^2^)					
≥1000	5	89 (79–95)	85 (69–93)	45 (13–160)	91 (89–94)
<1000	10	88 (79–93)	76 (64–85)	22 (12–41)	90 (87–92)
Biomarker					
ΔADC	10	88 (75–94)	80 (71–87)	29 (10–83)	91 (88–93)
Pre-NAC ADC	6	90 (74–96)	63 (52–73)	15 (5–41)	79 (75–82)
Post-NAC ADC	5	91 (78–96)	78 (58–90)	34 (13–87)	92 (90–95)
Study design					
Retrospective	8	91 (80–96)	75 (60–86)	30 (12–77)	92 (89–94)
Prospective	4	82 (71–84)	76 (66–84)	15 (7–34)	86 (83–89)
Magnetic field					
1.5T	9	91 (83–95)	77 (63–87)	33 (13–80)	93 (90–95)
3.0T	6	85 (70–91)	82 (71–89)	22 (10–47)	89 (86–91)
Model					
ADC	13	88 (81–93)	79 (69–87)	27 (15–50)	91 (88–93)
IVIM	2	86 (64–97)	76 (63–87)	14 (3–60)	NA

Note: Numbers in parentheses are the 95% CIs. NA = not applicable.

**Figure 5:**
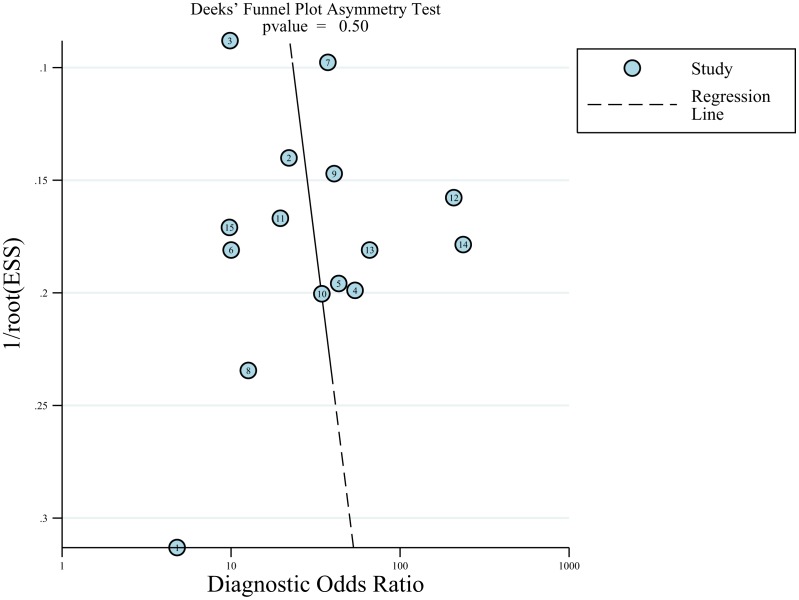
Funnel plot of publication bias The results showed no evidence of notable publication bias (*P* = 0.50).

The results of the Deeks funnel plot asymmetry test showed no evidence of notable publication bias (P = 0.50); see in Figure 5.

**Figure 6:**
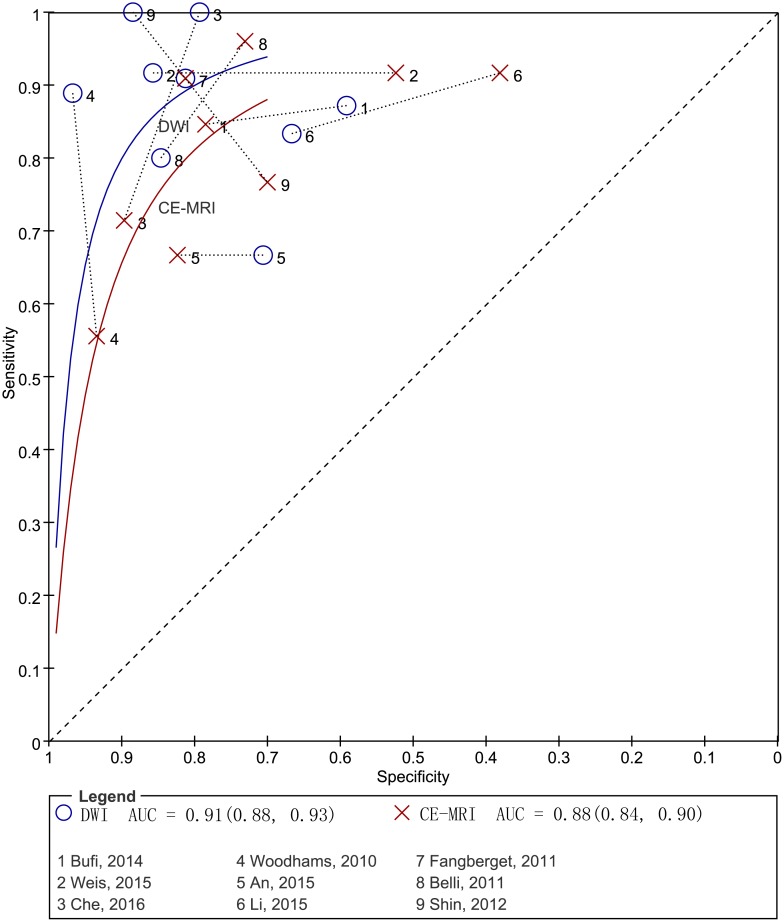
Pairs of observed sensitivity and specificity values for DWI and CE-MRI in HSROC curves

To compare the accuracy between DWI and CE-MRI effectively, we performed a pooled analysis using head-to-head comparative diagnostic accuracy studies (Table 4, Figure 6). The pooled weighted values for DWI were a SEN of 0.89 (95% CI: 0.81, 0. 93), a SPE of 0.81 (95% CI: 0.71, 0.89), a DOR of 33.72 (95% CI: 13.93, 81.59), and an AUC of 0.91 (95% CI: 0.88, 0.93). The pooled weighted values for CE-MRI were a SEN of 0.84 (95% CI: 0.74, 0. 91), a SPE of 0.76 (95% CI: 0.64, 0.85), a DOR of 16.57 (95% CI: 9.80, 28.02), and an AUC of 0.88 (95% CI: 0.84, 0.90).

**Table 4:** Summary of meta-analyses focused on CE-MRI and DWI for the assessment of breast cancer responses to NAC

Study	Search date	Comparative	No.	Technique	PSEN(95% CI)	PSPE(95% CI)	DOR(95% CI)	rDOR	AUC (95% CI)
Wu [30]	2000 to 2012	Indirect comparative	30	CE-MRI	0.68 (0.57, 0.77)	0.91 (0.87, 0.94)	55.59 (21.80, 141.80)		NR
			6	DWI	0.93 (0.82, 0.97)	0.79 (0.74, 0.83)	20.99 (13.24, 33.25)	0.38	NR
Liu [31]	1992–2015	Indirect comparative	54	CE-MRI	0.68 (0.66, 0.78)	0.84 (0.80, 0.88)	13.82 (7.28,26.23)		0.88 (NR)
			8	DWI	0.79 (0.68, 0.88)	0.75 (0.70, 0.80)	18.68 (6.88–50.73	1.35	0.87 (NR)
Our	2000 to 2017	Direct comparative	9	CE-MRI	0.84 (0.74, 0.91)	0.76(0.64, 0.85)	16.57 (9.80, 28.02)		0.88(0.84, 0.90)
			9	DWI	0.89 (0.81, 0.93)	0.81(0.71, 0.89)	33.72 (13.93, 81.59)	2.04	0.91 (0.88, 0.93)

Notes: No.= no. of studies; PSEN = pooled sensitivities; PSPE = pooled specificities; DOR = diagnostic odds ratio; AUC= areas under the ROC curve; DWI = diffusion weighted imaging; CE-MRI = contrast-enhanced MRI; rDOR = the ratio of DOR value of DWI divided by DOR value of CE-MRI; NR = not reported.

## DISCUSSION

Although breast MRI has been recommended as a clinical tool for NAC response evaluation for operable breast cancer, DWI has emerged as a potential imaging modality providing an early response biomarker based on ADC [26]. In this meta-analysis, we aimed to provide an overview of current strengths and weaknesses of DWI and to evaluate its accuracy for predicting the pCR to NAC in breast cancer using available data. The AUC of 15 studies concerning the new modality for estimating the pCR after NAC in breast cancer was 0.91 (95% CI: 0.88, 0.93), which indicated good diagnostic performance. However, the homogeneity test of sensitivity and specificity showed notable heterogeneity. The threshold effect might be a source of heterogeneity, as most of the studies included in the present analysis set a threshold but did not pre-specify the threshold. The results of threshold effect assessment indicated that the threshold effect was indeed the most important factor and likely contributed to 95% of the heterogeneity.

In addition to the threshold effect, certain putative factors might enhance heterogeneity, for example the choice of the b value may affect the ADC calculated by multiple pools diffusing at different rates [27]. The ADCs tend to be higher due to the contribution of perfusion in low b values and may also be preferable for differentiating malignant from benign tissues exclusively based on their water diffusion characteristics at high b values. However, the higher the b value is, the worse the signal-to-noise ratio becomes; this relationship restricts the clinical application of a high b value. To date, there is no consensus regarding the optimal b value in DWI studies [9]. In our subgroup analysis (Table 3), the results demonstrated that higher b value subgroups might outperform lower b value subgroups in assessing the pCR to NAC, with a higher pooled specificity (0.85 vs. 0.76) and a comparable pooled sensitivity (0.89 vs. 0.88). However, there were no notable differences between these two subgroups.

It has been shown that the change in diffusion coefficients is inversely correlated with therapeutic responses, and several studies [11–13, 18, 19, 23, 24] have noted that the change in ADC is an optimal biomarker for predicting the pCR in breast cancer. However, some studies [15, 21, 25] have shown that the pre-NAC ADC value is higher in subjects achieving a pCR compared with those showing residual disease, while other studies [17, 20] have suggested using post-NAC ADC. Therefore, we performed a subgroup analysis of different biomarkers of DWI. The performance of the ΔADC subgroup appeared to be equivalent to that of the post-NAC ADC subgroup, with a comparable pooled sensitivity and pooled specificity. Moreover, both subgroups had a higher pooled specificity than did the pre-NAC ADC subgroup (0.80 vs. 0.63). Thus, the breast cancer cells of responders are reduced and become necrotic in the form of a sieve during NAC, resulting in more significant changes in the diffusion parameters than observed for the breast cancer cells of non-responders.

Using a traditional monoexponential model, the ADC value can be calculated and used to quantitatively reflect the diffusion of water molecules in cancer tissue [27]. However, both pure molecular diffusion and perfusion in microcapillary circulation contribute to the ADC value. This contribution weakens the ability of ADC to characterise tissue microstructure [25]. Therefore, an IVIM model was developed to separate molecular diffusion from perfusion by using a wide range of low and high b values [28]. Only 2 of the 15 studies included in our meta-analysis followed the IVIM model. Che *et al.* [14] demonstrated that the best biomarker was the change in the true molecular diffusion coefficient (D), which yielded a high sensitivity of 1.00 and a specificity of 0.79. Bedair *et al.* [25] further suggested that the pre-treatment distributed diffusion coefficient (DDC) could potentially predict the pCR in breast cancer treatment with a sensitivity of 0.79 and a specificity of 0.73. In our subgroup analysis, we found that the IVIM model subgroup appeared to be equivalent to the ADC model subgroup, with a similar pooled sensitivity (0.86 vs. 0.88) and pooled specificity (0.76 vs. 0.79). Based on the small number and sample size of the studies, additional clinical trials focused on the ability IVIM to predict the pCR in breast cancer treatment are required.

Classically, the response to NAC has been identified by CE-MRI alone with Response Evaluation Criteria in Solid Tumours (RECIST) during NAC [29]. Compared with CE-MRI, DWI is able to obtain both anatomic and functional information simultaneously. Two previous meta-analyses [30, 31] compared the accuracy of DWI and CE-MRI for indirectly evaluating the pCR to breast cancer NAC, with only 6 [30] and 8 [31] studies available. Both meta-analyses congruously reported that DWI had a higher pooled sensitivity but a lower pooled specificity than CE-MRI. As direct comparisons provide the best effects of the diagnostic test accuracy of the two techniques [32, 33], we included exclusively head-to-head comparative studies that evaluated these two techniques in the same cohort. Our results showed that the pooled sensitivity, specificity and AUC of DWI were slightly higher than those of CE-MRI, in contrast to the results of the previous studies. Based on the data of the DOR value shown in Table 4, we observed an underlying trend that DWI is increasingly superior to CE-MRI with an increase in the number of DWI studies.

Some intrinsic limitations should be considered in the present study. First, because different tumour subtypes of breast cancer receive different NAC regimens with different histopathological responses, a quantitative analysis based on tumour subtype is highly desirable. However, only two studies [17, 21] on breast cancer subtypes were identified in the present meta-analysis. Liu *et al.* [17] showed that post-NAC ADC appears to be a promising tool for determining the pCR to NAC in breast cancer subtypes. The AUCs of the luminal A, luminal B, HER2-enriched, and triple-negative subtypes were 0.86, 0.86, 0.79, and 0.75, respectively. However, Richard *et al.* [21] found that the pre-NAC ADC of the triple-negative subtype was significantly higher in non-responders than in the pCR group, but no significant differences were observed in the luminal A and B subtypes. Second, diagnostic test accuracy estimates could also be influenced by the definition of pCR [34]. As there were too many pCR definitions (e.g., Chevallier-Sataloff classification, Miller-Payne Grading System, Mandard's TRG classification, RCB Index, or classification by user-defined) to perform a subgroup analysis, we did not assess pCR in our analyses. Third, there is notable heterogeneity in this meta-analysis. Many other factors, such as standards of DWI measurement, analysis, and cutoff values of diagnosis in DWI techniques, should be investigated. However, no consensus has been reached regarding those standards, making it difficult to summarise these factors in a meta-analysis.

In summary, this meta-analysis, which included 15 studies and 1081 patients, showed that DWI may be an accurate and nonradioactive imaging technique and might even be superior to conventional CE-MRI with respect to identifying the pCR to NAC in breast cancer. However, considering the notable heterogeneity and existing inherent limitations, this technique should be applied cautiously. In addition, there are a variety of issues concerning the assessment of DWI techniques for estimating breast cancer responses to NAC. Therefore, using a clear pCR definition, appropriate standards of DWI measurement, analysis, cutoff values, and large scale/multi-centred and well-designed clinical trials are necessary to assess the diagnostic value of DWI in breast NAC.

## MATERIALS AND METHODS

We employed the Preferred Reporting Items for Systematic Reviews and Meta-Analyses statement [35] to enhance the reporting of the present study (Figure 1).

### Search strategy

A structured approach was followed to detect the patient population, interventions, comparators, outcomes, and study design (PICOS criteria) [35]. Two authors searched the data sources (PUBMED, EMBASE, Web of Science, and the Cochrane Library) independently. The search strategy (Appendix A) comprised both subject headings (MeSH terms) and keywords for the target condition (breast cancer), the imaging under investigation (DWI), and the interventions (neoadjuvant therapy). We limited our search to studies published no later than July 2017. Review articles, letters, comments, case reports, and unpublished articles were excluded. Extensive cross-checking of the references in all the retrieved articles was performed.

### Criteria for inclusion in the study

Studies were considered available if the following PICOS criteria were met: (a) the patient population consisted of primary breast cancer confirmed histologically, (b) the imaging response to NAC was assessed using DWI, (c) histopathologic analysis was eligible as a gold standard, (d) a pCR or a near-pCR to NAC was described as an outcome and (e) both prospective and retrospective design were included.

We excluded studies if a 2 × 2 table could not be extracted from the data, if a full-text translation or evaluation for Non-English and non-Chinese articles could not be obtained, and if multiple reports were published for the same cohort. In the latter case, the most detailed or recent publication was extracted.

### Selection of articles

The selection of articles was performed independently by two authors, who initially screened the search results in titles and abstracts and further retrieved the full text of all potentially relevant reports. Next, the authors reviewed all relevant items according to the predefined inclusion criteria. Disagreements were arbitrated by a third author, who assessed all involved issues.

### Quality assessment and data extraction

For each included study, the methodological quality was evaluated independently by the three aforementioned authors, who extracted data from the selected reports using the standard quality assessment of diagnostic studies (QUADAS-2) items [36–38]. Additionally, associated data, including author, study nation, population and tumour characteristics, descriptions of definition of pCR and NAC regimens, study design, magnetic field strength, standards of DWI techniques, evaluation time, and descriptions of interpretations of the diagnostic tests, were also extracted from each study. The true-positive, false-positive, true-negative, and false-negative data were extracted and derived to construct 2×2 contingency tables.

### Meta-analysis

We constructed forest plots to demonstrate the variations of the sensitivity (SEN) and specificity (SPE) estimates together for DWI in each study and calculated the SEN, SPE, PLR, NLR and DOR values with 95% CIs. Hierarchical summary receiver operating characteristic (HSROC) curves were generated to assess SEN and SPE [39]. Standard χ^2^-testing and the inconsistency index (I-squared, I^2^) were used to estimate the heterogeneity of the individual studies using Stata software (version 14.0, Stata Corporation, College Station, TX, USA). If notable heterogeneities were detected (P < 0.1 or I^2^ > 50% [40]), the performance was pooled using a random-effects coefficient binary regression model; otherwise, a fixed-effects coefficient binary regression model was used [32]. Threshold effect testing, sensitivity analysis and meta-regression were used to explore heterogeneity.

The following subgroup analyses were carried out: (a) comparisons of studies using different b values: lower b value subgroup (≥ 1000 s/m^2^) or higher b value subgroup (< 1000 s/m^2^); (b) comparisons of studies with different biomarkers: ΔADC subgroup, pre-treatment subgroup or post-treatment subgroup; (c) comparisons of studies using a different magnetic field: 1.5 T subgroup or 3.0 T subgroup; (d) comparisons of studies with a different study design: retrospective subgroup or prospective subgroup; and (e) comparisons of studies using different diffusion models: ADC subgroup or IVIM subgroup.

Deeks funnel plots were generated and an asymmetry test was performed to assess publication bias. The existence of a nonzero slope coefficient (*P* < 0.05) was considered evidence of notable publication bias [41].

## SUPPLEMENTARY MATERIALS FIGURES AND TABLES





## Abbreviations

AUC: area under the ROC curve
ADC: apparent diffusion coefficient
CE: contrast enhanced
CI: confidence interval
DOR: diagnostic odds ratio
DWI: diffusion-weighted imaging
HSROC: hierarchical summary receiver operating characteristic
IVIM: intravoxel incoherent motion
MRI: magnetic resonance imaging
NAC: neoadjuvant chemotherapy
NLR: negative likelihood ratio
pCR: pathologic complete response
PLR: positive likelihood ratio
QUADAS: quality assessment of diagnostic studies
SEN: sensitivity
SPE: specificity.
