# Supplementary material for: Diffusion-weighted imaging in identifying breast cancer pathological response to neoadjuvant chemotherapy: A meta-analysis
Source: Oncotarget. 2017 Dec 11;9(6):7088–100. doi: 10.18632/oncotarget.23195 (PMC5805538; doi:10.18632/oncotarget.23195)
Supplement: Supplementary file 1 [file oncotarget-09-7088-s001.pdf]

Diffusion-weighted imaging in identifying breast cancer pathological response to neoadjuvant chemotherapy: A meta-analysis

SUPPLEMENTARY MATERIALS

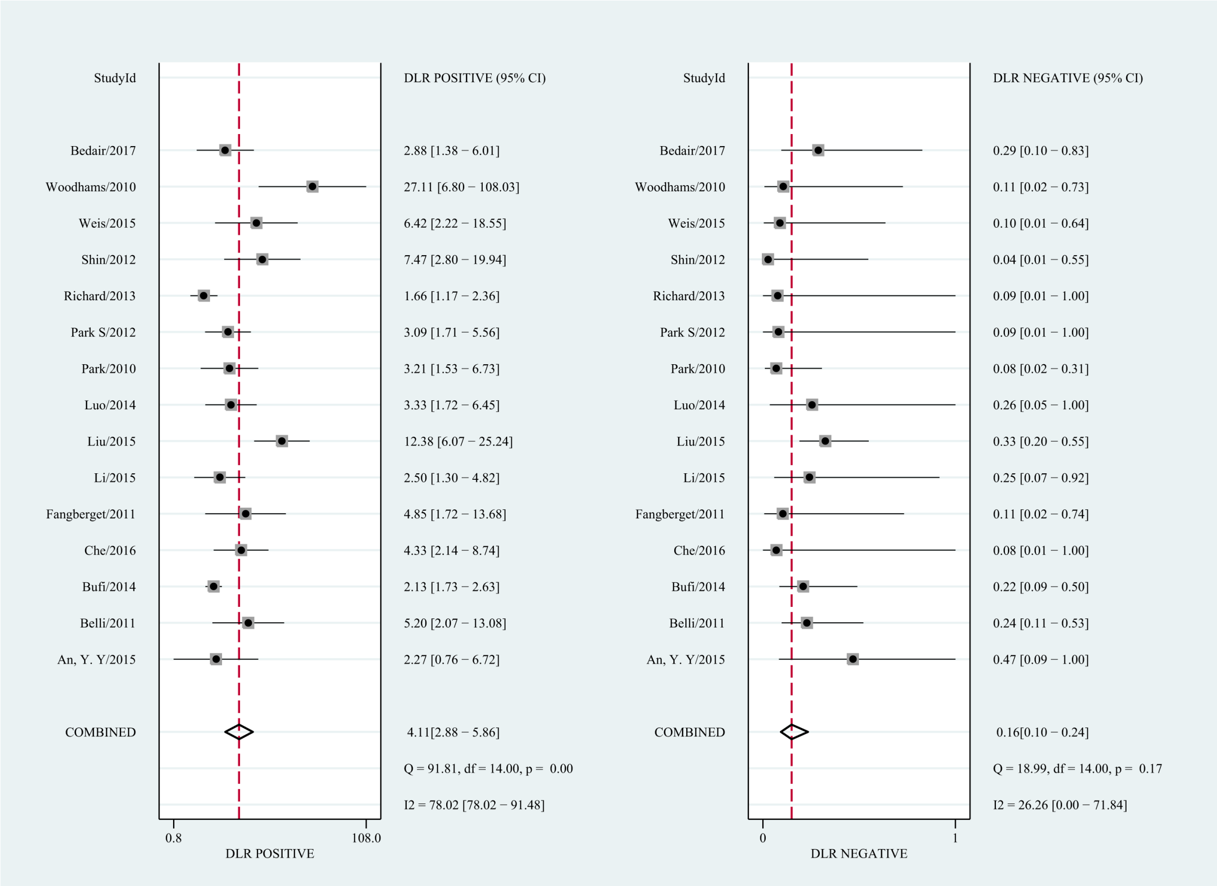

Supplementary Figure 1: Forest plots of the PLR and NLR and corresponding 95% CIs for DWI as an assessor of the pathologic response to NAC.

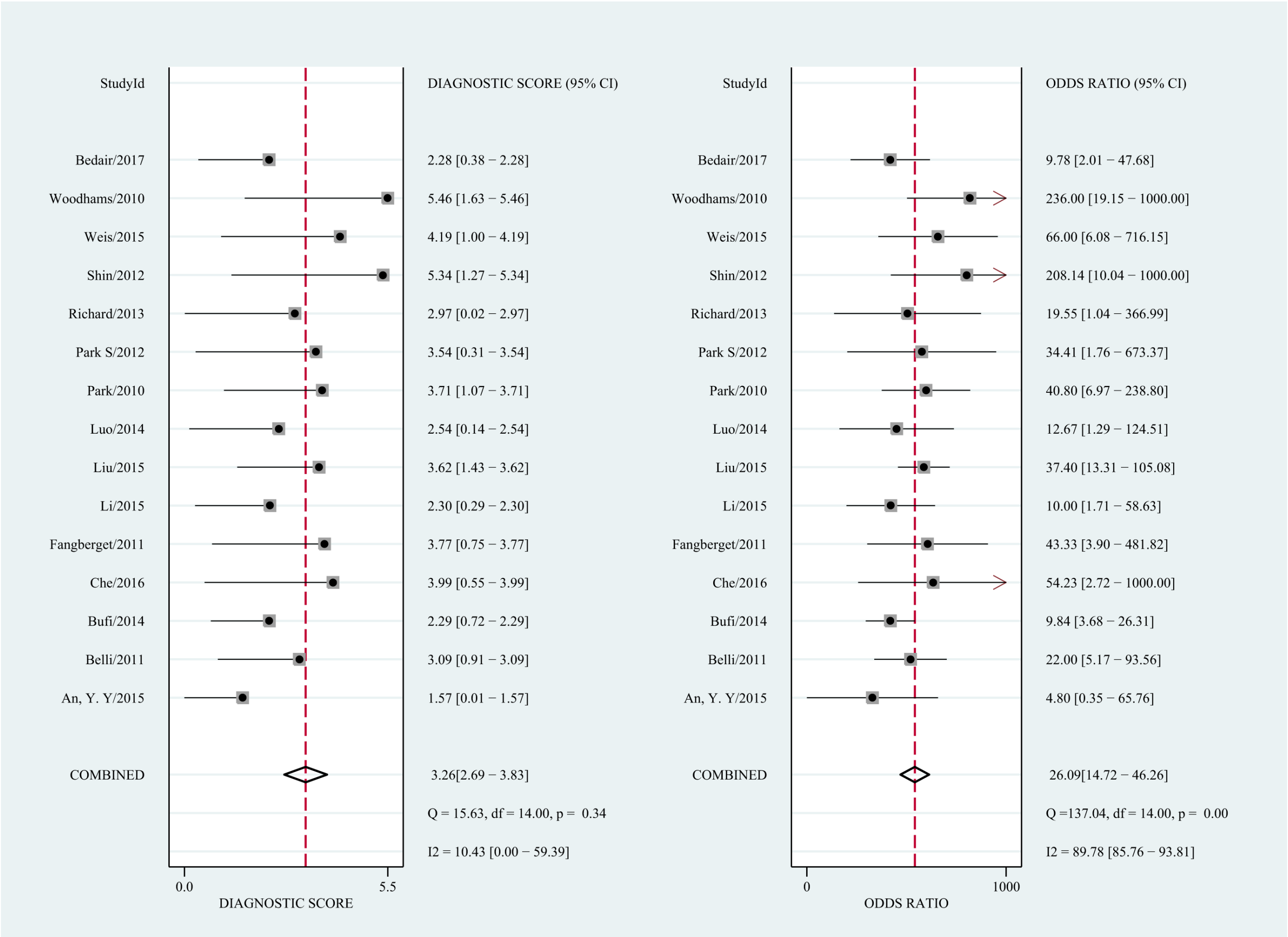

Supplementary Figure 2: Forest plots of the DOR and diagnostic score and corresponding 95% CIs for DWI as an assessor of the pathologic response to NAC.

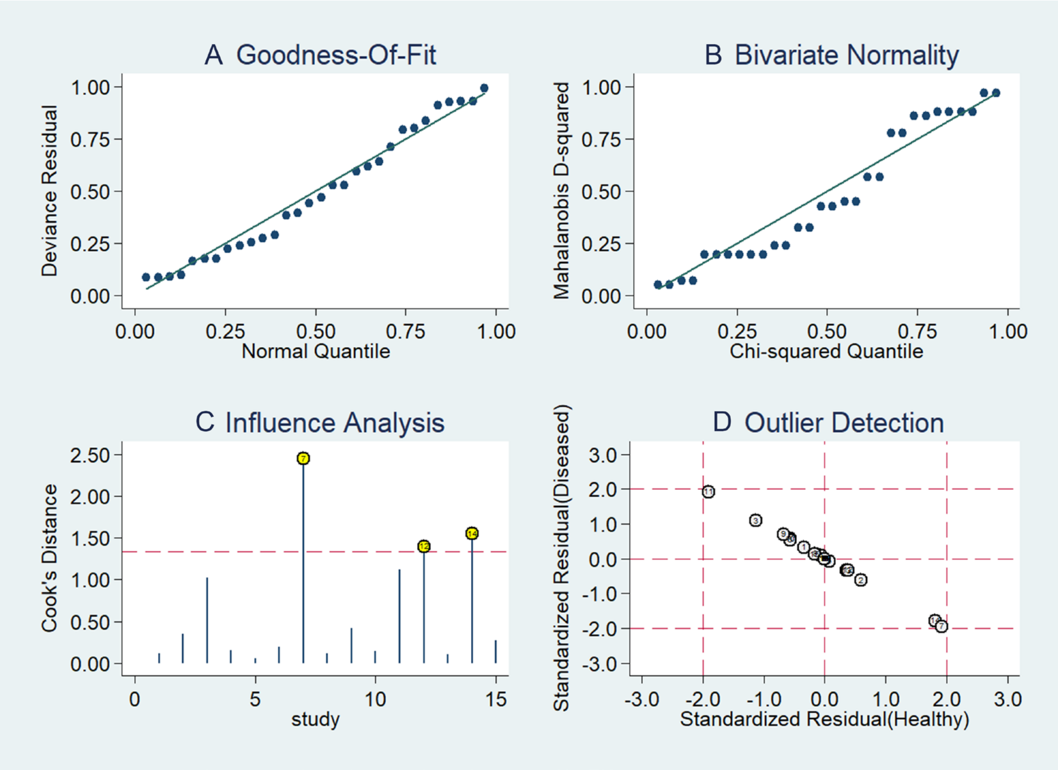

Supplementary Figure 3: Sensitivity analysis. Three studies were detected.

Supplementary Table 1: Summary of cohort, tumor stage, histologic type, treatment regimens, pathologic response classification and imaging characteristics of included studies*. See Supplementary_Table_1

Appendix_A

Search String

Free-Text Search String

(((((diffusion MR imaging) OR diffusion) OR DWI) OR diffusion weighted imaging) OR diffusion-weighted imaging) AND (((((((((breast neoplasm) OR breast tumor) OR Breast Cancer) OR breast carcinoma) OR mammary neoplasm) OR mammary carcinomas) OR mammary cancer) OR cancer of breast) OR malignant neoplasm of breast) OR malignant tumor of breast) cancer of the breast) AND (((neoadjuvant therapy) OR neoadjuvant chemotherapy) OR neoadjuvant treatment).

MeSH Search String

(((Diffusion Weighted Imaging [MeSH Terms]) AND Breast Neoplasms [MeSH Terms]) AND Neoadjuvant Therapy [MeSH Terms]).
