# Supplementary material for: Diffusion-weighted imaging in identifying breast cancer pathological response to neoadjuvant chemotherapy: A meta-analysis
Source: Oncotarget. 2017 Dec 11;9(6):7088–100. doi: 10.18632/oncotarget.23195 (PMC5805538; doi:10.18632/oncotarget.23195)
Supplement: Supplementary file 2 [file oncotarget-09-7088-s002.doc]

**Supplementary Table 1: Summary of cohort, tumor stage, histologic type, treatment regimens, pathologic response classification and imaging characteristics of included studies***

| **Study** | **Year** | **Stage(No.)** | **Histologic type (No.)** | **Receptor(No.)** | **NAC regimens (No.)** | **Pathologic response classification (No.)** | **MR Field** | **Modality of MR (Parameter)** | **b value** |
| --- | --- | --- | --- | --- | --- | --- | --- | --- | --- |
| **An, Y** | **2015** | II (2), III (18) | IDC (19), ILC (1) | LA (5), LB (7),TN (3), HER2+ (5) | [Dox+Doc](13), [Dox+Cyc](6), [(Dox+Doc)/(Dox+Cyc)] (1) | RCB Index: (0,1)=responder, (2,3)=nonresponder | Siemens 1.5T | CE-MRI(ΔLD), DWI(ΔADC) | 0,750 |
| **Belli** | **2011** | NR | IDC(38), ILC(7), Mixed(2), Other(4) | NR | [FEC](6), [Dox+Tax](23), [Tax+Dox+Cyc](9), [(Tax++Cyc)+car/tra](13), all for 4-6 cycles | Mandard's TRG classification: (1-3)=responder, (4-5)=nonresponder | GE 1.5T | CE-MRI(ΔLD), DWI(ΔADC) | 0,1000 |
| **Bufi** | **2014** | (Ⅱ+Ⅲa)(180), [Ⅲb+Ⅲc+IV] (45) | IDC(177), ILC(36), other(12) | Luminal(143), TN(37), HER2+(17), Hybrid(28) | [(Tax++Cyc)2-4 + tax](225) | Mandard's TRG classification: 1=pCR (absenceofanyresidualdisease) | GE 1.5T | CE-MRI(ΔLD), DWI(ΔADC) | 0,1000 |
| **Che** | **2016** | II (7), III (29) | IDC (34), ILC (2) | LA (4), LB (26), HER2+(2), other (4) | Pac+Epi, Pac+Car, all for 4-8 cycles | Miller-Payne Grading System: 5=pCR, (1-4)=non-Pcr | GE 3.0T | DWI(ΔD*, D) | 0,800,1000 |
| **Fangberget** | **2011** | III (21) | IDC (24), ILC (7) | ER(+) (21), PR(+) (18), HER2+ (11), TN (4) | [FEC 6](10), [FEC 4+Tax 2](18), [FEC 3+Tax+Tra](2) | pCR and non-pCR (pCR: No invasive cancer, but DCIS may be present) | Siemens 3.0T | CE-MRI(ΔLD), DWI(ΔADC) | 100, 250, 800 |
| **Li** | **2015** | NR | NR | ER(+) (16), PR(+) (16), HER2+ (12), TN (12) | [Dox+Cyc+Tax](20), [Cis/Tax+/−RAD001](10), [Tra+Car](3), [Tra/Car/Her](3), [Tax](1) | pCR and non-pCR (pCR: No invasive cancer) | Philips 3.0T | CE-MRI(ΔLD), DWI (ΔADC, ADC) | 0,600 |
| **Liu** | **2015** | IIb (30), III (146) | IDC(156), ILC(13), other(7) | LA (67), LB (45), HER2+ (29), TN (35) | [(Dox+Cyc)4+Doc 4](176) | Miller-Payne Grading System: (4-5)=pCR, (1-3)=non-pCR | Philips 3.0T | DWI(ΔADC) | 0,800 |
| **Luo** | **2014** | NR | NR | NR | NR | Miller-Payne Grading System: (4-5)=pCR, (1-3)=non-pCR | Philips 3.0T | DWI(ΔADC) | 0,800 |
| **Park** | **2010** | II (27), III (26) | IDC (35), Mixed (1) | ER(+) (30) PR(+) (25) HER2+ (9) | [(Dox+Doc) 3] after granulocyte colony-stimulating factor | pCR and non-pCR (pCR: No invasive cancer, but DCIS may be present) | GE 1.5T | CE-MRI(ΔLD), DWI(ADC) | 0,750 |
| **Park S** | **2012** | NR | IDC (32), MC (1), Mixed (1) | NR | [Dox+Doc 3] (23), [Pac+Gem+Tra 6] (11) | pCR and non-pCR (pCR: No invasive cancer, but DCIS may be present) | GE 1.5T | DWI(ΔADC) | 0,750 |
| **Richard** | **2013** | II (33), III (63), IV(22) | IDC(109), ILC(6), other(3) | LA (33), LB (28), HER2+ (11), TN (37) | EC, EC+Doc, EC+Tra+Doc | Chevallier-Sataloff classification: (1 or A)=pCR, (2-4 or B,C,D)=non-pCR | Siemens 1.5T | DWI(ADC) | 50,700 |
| **Shin** | **2012** | IIa(30), IIb(27), IIIa (22), IIIb(3), IIIc(8) | IDC(83), ILC(3), MC(3), other (1) | ER(+) (41), PR(+) (64), HER2+ (64) | [Doc+Cyc 4](9), [Dox(Adr)+Cyc 4](67), [Adr+Doc] (8), FEC(6), [Tra+Pac 4 or 6] (5) | pCR and non-pCR (pCR: No invasive cancer, but DCIS may be present) | Siemens 1.5T | DWI(ΔADC), MRS(ΔtCho) | 0,100,500,800,1000 |
| **Weis** | **2015** | II, III | NR | ER(+) (11), PR(+) (11), HER2+ (12), TN (10) | [AC+Tax](16), [AC+Tax+Tra](3), [Tax+Cis+Eve] (8), [Tax+Car+Her] (3), [Tra+Lap](2), Tax(1) | pCR and non-pCR (pCR: No residual viable tumor) | Philips 3.0T | CE-MRI(ΔLD), DWI(ΔADC) | 0,500 or 600 |
| **Woodhams** | **2010** | NR | IDC(62), ILC(6), other (2) | NR | [(Ant+Cyc)4+Pac 4] | pCR and non-pCR (pCR: No residual viable tumor) | GE 1.5T | CE-MRI(ΔLD), DWI(ΔADC) | 0,1500 |
| **Bedair** | **2017** | II (17), III (19) | IDC(30), ILC(4), other (2) | ER(+) (24), HER2+ (13) | [(Doc 3 + FEC 3] | pCR and non-pCR (pCR: No invasive cancer, but DCIS may be present) | GE 3.0T | DWI(DDC, ADC) | 0, 60,120, 300, 600, 900 |

* Abbreviations: AC, doxorubicin+cyclophosphamide; Adeno, adenocarcinoma; Adr, adriamycin; Ant, anthracycline; Cap, capecitabine; Car, carboplatin; Cis, Cisplatin; Cyc, cyclophosphamide; D*, perfusion-related diffusion; DCIS, ductal carcinoma in situ; DDC, distributed diffusion coefficient; Doc, docetaxel; Dox, doxorubicin; Epi, epirubicin; ER, estrogen receptor; EC, epirubicin+cyclophosphamide; Eve, everolimus; FEC, 5-flourouracil+epirubicin+cyclophosphamide; Gem, gemcitabine; Her, herceptin; HER2, humanepidermal growth factor receptor 2; IDC, invasive ductal carcinoma; ILC, invasive lobular carcinoma; IMC, invasive micropapilary carcinoma; LA, luminal A; LABC, locally advanced breast cancer; Lap, lapatinib; LB, luminal B; MRD, minimal residual disease; MC, mucinous carcinoma; NAC, neoadjuvant chemotherapy; NR, not reported; Pac, paclitaxel; pCR, pathologic complete response; PR, progesterone receptor; RCB, residual cancer burden; Tax, taxane; Tra, trastuzumab; ΔLD, change in longest diameter; ΔADC, change in apparent diffusion coefficient; ΔKep, change in transfer constant; ΔtCho, change in total choline-containing compounds.
